# Estrogens, selective estrogen receptor modulators, and a selective estrogen receptor down-regulator inhibit endothelial production of tissue factor pathway inhibitor 1

**DOI:** 10.1186/1471-2261-6-40

**Published:** 2006-10-09

**Authors:** Anders EA Dahm, Nina Iversen, Baard Birkenes, Anne Hansen Ree, Per Morten Sandset

**Affiliations:** 1 Department of Haematology, Ullevål University Hospital, 0407 Oslo, Norway; 2 Faculty Division Ullevål, University of Oslo, 0407 Oslo, Norway; 3 Department of Medical Genetics, Ullevaal University Hospital, 0407 Oslo, Norway; 4 Department of Tumor Biology and Department of Oncology, Rikshospitalet-Radiumhospitalet Medical Center, 0310 Oslo, Norway; 5 Faculty Division Rikshospitalet-Radiumhospitalet, University of Oslo, Oslo, Norway

## Abstract

**Background:**

Hormone therapy, oral contraceptives, and tamoxifen increase the risk of thrombotic disease. These compounds also reduce plasma content of tissue factor pathway inhibitor-1 (TFPI), which is the physiological inhibitor of the tissue factor pathway of coagulation. The current aim was to study if estrogens and estrogen receptor (ER) modulators may inhibit TFPI production in cultured endothelial cells and, if so, identify possible mechanisms involved.

**Methods:**

Human endothelial cell cultures were treated with 17β-estradiol (E2), 17α-ethinylestradiol (EE2), tamoxifen, raloxifene, or fulvestrant. Protein levels of TFPI in cell media and cell lysates were measured by an enzyme-linked immunosorbent assay, and TFPI mRNA levels were assessed by quantitative PCR. Expression of ERα was analysed by immunostaining.

**Results:**

All compounds (each in a concentration of 10 nM) reduced TFPI in cell medium, by 34% (E2), 21% (EE2), 16% (tamoxifen), and 28% (raloxifene), respectively, with identical inhibitory effects on cellular TFPI levels. Expression of TFPI mRNA was principally unchanged. Treatment with fulvestrant, which was also associated with down-regulation of secreted TFPI (9% with 10 nM and 26% with 1000 nM), abolished the TFPI-inhibiting effect of raloxifene, but not of the other compounds. Notably, the combination of 1000 nM fulvestrant and 10 nM raloxifene increased TFPI secretion, and, conversely, 10 nM of either tamoxifen or raloxifene seemed to partly (tamoxifen) or fully (raloxifene) counteract the inhibitory effect of 1000 nM fulvestrant. The cells did not express the regular nuclear 66 kDa ERα, but instead a 45 kDa ERα, which was not regulated by estrogens or ER modulators.

**Conclusion:**

E2, EE2, tamoxifen, raloxifene, and fulvestrant inhibited endothelial production of TFPI by a mechanism apparently independent of TFPI transcription.

## Background

Hormone therapy (HT) [1-4], oral contraceptives (OCs) [5,6], raloxifene[7] and tamoxifen[8] are all associated with increased risk of venous thromboembolism (VTE). HT [9] and OCs [10,11] also increase the risk of arterial thrombosis. The mechanism(s) by which these drugs may cause thrombotic diseases is not fully understood. They are thought to induce "acquired" resistance to activated protein C[12,13] and also change plasma contents of several coagulation factors and coagulation inhibitors, which assumingly may alter the "haemostatic balance" in a prothrombotic direction [14,15].

The therapeutic action of HT, OCs, raloxifene, and tamoxifen is mediated by the estrogen receptor-α (ERα). The estrogen component of OCs is 17α-ethinylestradiol (EE2), whereas HT contains either 17β-estradiol (E2) or conjugated equine estrogens. While E2 and EE2 are strong ER agonists, raloxifene and tamoxifen are defined as selective ER modulators (SERMs). Raloxifene is a relatively new drug for treatment of osteoporosis, whereas tamoxifen for several decades has been used as first-line anti-hormonal therapy for ER-positive breast cancer in premenopausal patients. These compounds show tissue-specific agonistic or antagonistic effects. Recently, fulvestrant was approved as a novel breast cancer anti-estrogen. This compound belongs to a class of drugs termed selective ER down-regulators (SERDs), which have been claimed to be completely devoid of agonistic effects[16].

Tissue factor pathway inhibitor-1 (TFPI) is mainly produced by the endothelium [17] and is the physiological inhibitor of the tissue factor pathway of blood coagulation. This pathway is considered to be the principal initiator of coagulation [18]. TFPI binds both to activated factor X (FXa) and the activated factor VII (FVIIa)/tissue factor complex to build an inert quaternary complex, and thereby inhibits FXa as well as the TF/FVIIa complex. Low levels of circulating TFPI in plasma have been shown to double the risk for both venous[19] and arterial[20] thrombosis. Moreover, TFPI may be involved in angiogenesis[21] and contribute to the anti-angiogenic properties of heparins[22].

Tamoxifen[23], HT [24], and OCs[19,25] all reduce plasma concentration of TFPI. Furthermore, TFPI levels in premenopausal women are lower than in men and postmenopausal women. This suggests that estrogens or drugs mimicking effects of estrogens may reduce the production of TFPI, either by a direct effect on the endothelium or indirectly through other mechanisms.

The mechanism by which ER-binding drugs regulate TFPI is largely unknown. We hypothesized that E2, EE2, raloxifene, and tamoxifen might inhibit endothelial TFPI production by an ER-mediated genomic mechanism (via inhibition of TFPI mRNA expression) and that fulvestrant might counteract any inhibitory effect, i.e., that the effects on TFPI production might be classically ER-dependent. However, all tested compounds (estrogens, SERMs, and, interestingly, also fulvestrant) were found to down-regulate endothelial TFPI production, both in cell medium and in whole cell lysates, by a mechanism apparently independent of TFPI transcription.

## Methods

### Reagents

Before start of experiments, cells were cultured in growth medium containing EBM-2 basal medium (#CC-3156) supplemented with EGM-2-MV SingleQuots and growth factors (#CC-4147) in addition to 10% FCS (#DE14-801F). The experiments were performed in hormone-deficient medium containing EBM phenol red-free medium (#CC-3129) with 10% charcoal/dextran-stripped FCS (#14-820F), and supplemented with EGM-2 MV SingleQuots and growth factors (#CC-4147) minus hydrocortisone. All cell media and FCS were purchased from Cambrex Bio Science, Walkersville, MD, USA. E2 (#E2758), EE2 (#E4876), raloxifene hydrochloride (#R1402) and tamoxifen (#T5648) were purchased from Sigma. Fulvestrant (#1047), also termed ICI 182,780, was purchased from Tocris BioScience, Ellisville, MO, USA.

### Cell cultures and study design

Human coronary artery endothelial cells (HCAEC, #CC-2585) that had been isolated from single-donor, normal human tissue and cryopreserved in endothelial cell media, were purchased from Cambrex Bio Science, Walkersville, MD, USA. In our experiments, cells from both female and male donors were used. The cells were cultured in growth medium for three days, then trypsinated onto a 24-well plate and left overnight in growth medium before experimental procedures. At this stage the HCAEC were at passage seven. Human umbilical cord vascular endothelial cells (HUVEC) were isolated from veins of umbilical cords obtained from newborns after normal pregnancies, according to the method of Jaffe *et al*[26]. Isolated single-donor HUVEC were immediately seeded onto 24-well plates and left overnight in growth medium before experiments were performed, i.e., the HUVEC were at passage one.

Experimental procedures: HUVEC were preincubated in triplicates with hormone-deficient medium only (controls) or with hormone-deficient medium containing E2 (0.1–10 nM) for 48 h. Similarly, HCAEC were preincubated in triplicates for 48 h with hormone-deficient medium only (controls) or with hormone-deficient medium containing E2, EE2, tamoxifen, raloxifene, or fulvestrant (each 10 nM). To study any counteracting effects of SERD, fulvestrant (1000 nM) was added to control medium or the media containing each of the other compounds. After the preincubation, when the cells were 80% confluent, the experimental media were exchanged to new drug-containing media for additional 24 h before cell harvesting, i.e., the total incubation time in experimental media was 72 h.

### Assays of TFPI antigen and total protein

TFPI total antigen was assayed in duplicates with a commercial enzyme-linked immunosorbent assay (Asserachrom^® ^Total TFPI, Diagnostica Stago, Asnière, France) described in detail elsewhere[19]. Cellular total protein was measured by an improved Lowry assay (Bio-Rad *D*_*C *_Protein Assay, Bio-Rad Laboratories, Hercules, CA, USA). Levels of TFPI total antigen secreted into cell media and in whole cell lysates were normalised against the cellular total protein content of each sample.

### Relative quantification of TFPI mRNA

Cellular TFPI mRNA levels were determined by quantitative real-time RT-PCR (qRT-PCR) analysis (ABI PRISM 7700 Sequence Detection System, Applied Biosystems, Foster City, CA, USA). Primers and probes were designed using Primer Express software version 2.0 (Applied Biosystems, Foster City, CA, USA) and purchased from Medprobe (Eurogentec, 4102 Seraing, Belgium). Their sequences were: 5'-ACACACAATTATCACAGATACGGAGTT-3' (forward primer), 5'-TTCAAGGCGGATGATGGC-3' (reverse primer) and 5'-CCACCACTGAAACTTATGCATTCATTTTGTGC-3' (probe). Total RNA was isolated from lysed cells using the ABI PRISM 6100 Nucleic Acid Prep Station (Applied Biosystems, Foster City, CA, USA) according to the manufacturer's recommendations. The procedure included a step with DNAse I treatment (Applied Biosystems, Foster City, CA, USA). Total RNA was quantified by ND-1000 (NanoDrop Technologies, Delaware, USA), and RNA quality was checked by running an aliquot at RNA 6000 Nano LabChip^® ^on the 2100 Bioanalyzer (Agilent Technologies, CA, USA). 100–300 ng of total RNA were reversely transcribed using pd(N)_6 _random primers (First strand cDNA synthesis kit, Amersham Biosciences, Little Chalfont Buckinghamshire, Germany). qRT-PCR was performed using the TaqMan^® ^Universal Master mix (Applied Biosystems, Foster City, CA, USA) containing 2 μL cDNA, 300 nM of each primer and 200 nM probe in a 25 μL final volume. The reactions were carried out in triplicate in MicroAmp Optical 96-well plates covered with MicroAmp Optical Caps (Applied Biosystems, Foster City, CA, USA). The PCR program was 50°C for 2 min, 95°C for 10 min, 40 cycles of 95°C for 15 sec, and 60°C for 1 min. Relative mRNA levels were obtained by the comparative threshold method (2^-ΔΔCt method) (User bulletin no. 2, Applied Biosystems) and normalised to 18S RNA levels to compensate for variations in input of RNA amounts. Levels of 18S RNA was not affected by any of the drugs in the experiments.

### Immunostaining

Cells grown in 6-well plates were either harvested when culturing in growth medium for three days was completed, or treated identically as cell cultures used for TFPI analyses. Cell pellets were lysed in 1 mL ice-cold lysis buffer (M-PER Mammalian Protein Extraction Reagent #78501, PIERCE, Rockford, IL, USA), to which 10 μL protease inhibitor cocktail (PIERCE, Rockford, IL, USA) and 10 μL 0.5 M EDTA #ED2SS (Sigma-Aldrich, St. Louis, MO, USA) were added, mixed and incubated for 60 min at 4°C. The lysates were cleared by centrifugation for 30 min. Aliquots of 30 μg of total protein were separated on an 8% SDS-polyacrylamide gel and analysed by standard Western blot technique. The membranes were immunostained with a mouse monoclonal antibody to ERα (SC-8002; Santa Cruz Biotechnology, Inc., Santa Cruz, CA, USA), and with normal nonspecific mouse IgG (SC-2025; Santa Cruz Biotechnology), and with an anti-α-tubulin antibody (CP06; Calbiochem/Merck Biosciences, Ltd., Nottingham, UK). Immune complexes were detected with horseradish peroxidase-coupled polyclonal rabbit anti-mouse IgG (P0260, Dako, Glostrup, Denmark), and enzyme activity was visualised with enzyme-linked chemiluminescence (Amersham Biosciences, Ltd., Buckinghamshire, UK).

Competition studies were performed with a blocking peptide (SC-8002P; Santa Cruz Biotechnology). The peptide was preincubated with the anti-ERα antibody (SC-8002) for 24 h in 4°C before use.

### Statistics

The protein results were expressed as mean and standard deviation (SD) and tested for statistical significance with Student's two-tailed t-test. The PCR results were expressed as mean and SD and tested for statistically significance with the Relative Expression Software Tool (REST^©^) [27]. p ≤ 0.05 was considered statistically significant.

## Results

### Down-regulation of TFPI by E2 in HUVEC

**Figure 1:**
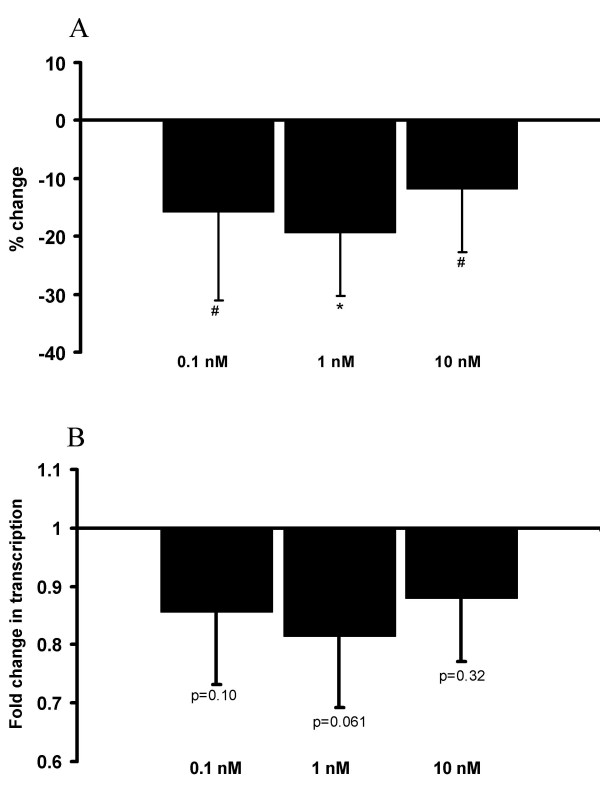
**Effects of E2 on secreted TFPI and TFPI mRNA expression in HUVEC**. The cells were incubated in the absence (controls) or presence of E2 (0.1–10 nM). Figure 1A indicates percent change (mean ± SD) of secreted TFPI in the media compared to controls, figure 1B shows fold change (mean ± SD) in TFPI mRNA compared to controls. The results are from two independent experiments. * is p < 0.01 and # is p = 0.05 compared to control wells.

HUVEC were incubated with increasing concentrations of E2 (0.1–10 nM) and TFPI production was analysed. As seen from Figure 1A, TFPI secreted to the medium was significantly reduced by 16% (0.1 nM E2), 20% (1 nM E2), and 12% (10 nM E2), respectively. Although TFPI mRNA expression was reduced by 14% (0.1 nM E2), 18% (1 nM E2), and 12% (10 nM E2) compared to control, these effects were not statistically different from the control condition (Figure 1B).

### Down-regulation of TFPI by ER ligands in HCAEC

**Figure 2:**
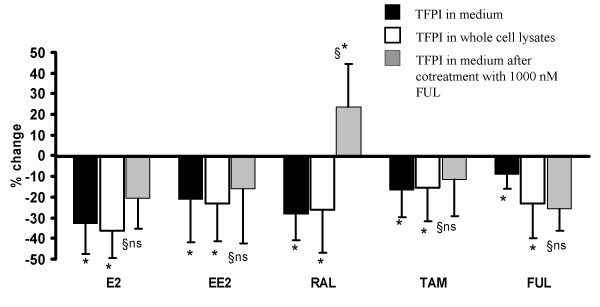
**Effects of ER ligands on TFPI production in HCAEC**. The cells were incubated in the absence (controls) or presence of 10 nM each of E2, EE2, raloxifene (RAL), tamoxifen (TAM) or fulvestrant (FUL). The bars show TFPI in the media (black bars), in whole cell lysates (white bars), or in the media after concurrent treatment with 1000 nM fulvestrant (gray bars) and indicate percent change (mean ± SD) compared to controls. Results are from three independent experiments. * is p ≤ 0.05. §refers to comparisons between respective gray and black bars, where §ns is non significant difference and §* is p ≤ 0.01

**Figure 3:**
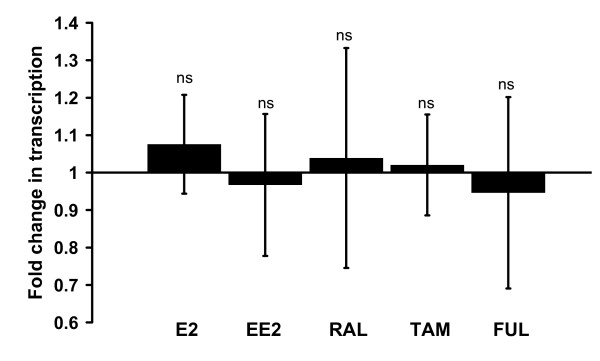
**Lack of ER ligand effects on TFPI-transcription**. The cells were incubated in the absence (controls) or presence of 10 nM each of E2, EE2, raloxifene (RAL), tamoxifen (TAM) or fulvestrant (FUL). The bars show mean (± SD) fold change in TFPI transcription in HCAEC. The results are from three independent experiments. ns is non significant compared to control wells.

In HCAEC, treatment with E2 (0.1–10 nM) reduced TFPI in the medium by 25% (0.1 nM), 26% (1 nM), and 20% (10 nM), but with no apparent change in transcription (data not shown). Furthermore, both estrogens (E2 and EE2) as well as SERMs (raloxifene and tamoxifen), each at a concentration of 10 nM, significantly inhibited TFPI protein production (Figure 2). Secreted TFPI was down-regulated by 34% (E2), 21% (EE2), 28% (raloxifene), and 16% (tamoxifen) compared to control cells, and corresponding results were obtained for cellular TFPI levels. The pure ER antagonistic compound fulvestrant was also included in this drug screen, and, notably, an inhibitory effect on the protein level of TFPI was observed. Compared to the control condition, 10 nM fulvestrant reduced TFPI secretion by 9%, whereas 1000 nM caused down-regulation by 26% (Figure 2). Again, TFPI mRNA expression in treated cells was not significantly different from that of control cells (Figure 3). In a single experiment, the effect of E2 (10 nM) on TFPI mRNA was followed every 8 h for a total period of 72 h, but without any convincing, timed-dependent change in the mRNA level (data not shown).

### Fulvestrant antagonised the inhibitory effect of raloxifene on TFPI

Fulvestrant acts by down-regulating ER expression and is principally devoid of agonistic effects. Hence, we investigated if fulvestrant might counteract the inhibition of TFPI production in HCAEC elicited by the other compounds. Compared with each of the other drugs alone (at concentrations of 10 nM), concurrent treatment with fulvestrant (1000 nM) fully antagonised the inhibitory effect of raloxifene, resulting in net increased TFPI secretion. The inhibitory effects of the other agents, however, were not significantly changed (Figure 2). On the other hand, 10 nM of tamoxifen and raloxifene seemed to fully (raloxifene) or partly (tamoxifen) antagonise the effects of 1000 nM fulvestrant alone (Figure 2, grey bars in TAM and RAL groups are significantly different from grey bar in FUL group).

### ERα of the human endothelial cell cultures

**Figure 4:**
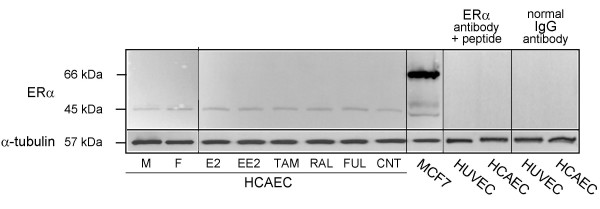
**ERα expression in HCAEC**. The Western blots depict ERα levels in HCAEC, isolated from a male (M) and a female (F) donor prior to initiation of ER ligand incubation as well as after completion of 72 h incubation in the absence (CNT) or presence of 10 nM each of E2, EE2, tamoxifen (TAM), raloxifene (RAL) or fulvestrant (FUL). A whole cell lysate of MCF7 cells was included as positive control and expression of α-tubulin was measured as loading control. Normal non-specific mouse IgG and a blocking peptide was included to ensure specificity of the bands.

Expression of ERα was analysed in both HCAEC and HUVEC before start and after completion of incubation with the various ER ligands. As seen from Figure 4, HCAEC were devoid of 66 kDa ERα, but instead expressed low levels of a 45 kDa ERα band both before and after drug treatment. Interestingly, the 45 kDa ERα was not regulated by any of the ligands. Normal IgG from mouse did not produce the 45 kDa band, and a competing peptide removed the 45 kDa band from the western blot. An identical 45 kDa ERα protein species was found in HUVEC before and after drug treatment (data not shown). No difference in ERα expression in the HCAEC from female or male donors was seen (Figure 4).

## Discussion

The present study showed that treatment of human endothelial cell cultures with E2, EE2, tamoxifen, raloxifene, or fulvestrant inhibited cellular TFPI production, by a mechanism that apparently did not involve TFPI transcription. These results experimentally support recently reported observations that tamoxifen, HT, and OCs reduce plasma concentration of TFPI in women [19,23-25]. To our knowledge, this report is the first to demonstrate that raloxifene and fulvestrant inhibit endothelial TFPI production, suggesting that also these compounds may cause lowering of plasma TFPI concentration upon therapeutic utilization.

Our results confirm the conclusion by Bilsel et al, who found E2-dependent reduction in secreted TFPI by HUVEC[28]. Moreover, Shirk et al observed that EE2 caused reduction in blood TFPI activity in rats, but did not manage to reproduce Bilsel et al's findings in HUVEC[29]. We also had difficulties in reproducing those results until we changed the study design to include 48 h preincubation with the compounds (see Material and Methods). The endothelial effect of OC, HT, and ER modulators is possibly the principal mechanism by which these compounds regulate plasma TFPI in women. Our results also suggest that the estrogen component of OCs and HT may be responsible for the TFPI-lowering effect of these drugs.

Fulvestrant has been claimed to be completely devoid of ER agonist effects[16]. Thus, it was somehow surprising to find that fulvestrant treatment, similar to what was observed with the other compounds, was associated with reduced TFPI production, although the equimolar fulvestrant concentration apparently showed weaker inhibitory activity. Indeed, concurrent treatment with a high concentration of fulvestrant seemed to fully antagonise the inhibitory effect of raloxifene, but not of the other ER ligands, on TFPI secretion. Conversely, tamoxifen and raloxifene, fully or partly, antagonised the effect of fulvestrant, i.e., SERMs and SERD antagonised each other. Shirk et al found that fulvestrant had no direct effect on TFPI in rats, but counteracted the TFPI-lowering effect of EE2[29]. In general, endothelial ER agonist effects are reversed by fulvestrant[30,31], although both E2, SERMs, and fulvestrant may act synergistically, e.g., by inhibiting neointimal thickening in rat [32]. It might be, at least in human blood coagulation, that fulvestrant exerts dual actions; i.e., agonistic when used as single agent but occasionally antagonistic to that function upon combinational treatment with SERMs.

TFPI levels in cell medium and whole cell lysates were similarly regulated by the various ER-ligands, which indicate that regulation does not involve cellular secretion of TFPI. Furthermore, cellular TFPI mRNA expression did not seem to be the target for ER-mediated regulation (although we cannot completely rule out a decrease of TFPI mRNA in HUVEC), which is consistent with lack of an estrogen responsive element in the human TFPI promoter[33] to mediate classical ER-directed responses. Our data are supported by Shirk et al, who did not find reduction in TFPI transcription, despite reduction in TFPI activity, in rats[29]. Since all compounds tested in our study are ER ligands, it is reasonable to assume that the effect on TFPI is mediated by ER. The cell cultures did not express the regular 66 kDa ERα, but a 45 kDa ERα. Hence, one might speculate that the regulation of TFPI involves post-transcriptional effect(s) mediated by the amino-terminally truncated 45 kDa version of ERα. This hypothesis is supported by studies showing that truncated ERα mediates non-genomic effects of ER ligands [34-36]. Alternatively, it might be that ERβ, or the recently described GPR30 [37], are involved in this regulatory pathway.

ER-modifying agents are shown to exert differential effects on stability of their 66 kDa receptor protein[38,39]. Such responses, termed autologous receptor regulation[40], are dependent on intact DNA-binding function of the amino-terminal ERα domain[41]. The observed absence of ligand-operated regulation of the endothelial amino-terminal truncated 45 kDa ERα is in accordance with this concept.

Moreover, the variety of biological effects elicited by ER-binding ligands are also governed by tissue-specific expression of ER cofactors and modifying signalling pathways implicated[42]. Within blood coagulation, indeed, estrogen agonists and ER modulators might differentially regulate endothelial expression and activation of factors like thrombomodulin and plasminogen activator inhibitor type 1 [43-45]. Unlike for SERMs [46], an unfavourable coagulation profile of fulvestrant has so far not been reported.

## Conclusion

The main findings in this study were that estrogens and ER modulators, including fulvestrant, reduced endothelial TFPI production possibly in absence of transcriptional or secretional down-regulation of TFPI. Furthermore, fulvestrant seemed to counteract the inhibitory effect of raloxifene but not of the other compounds tested. Since our cells did not express the regular 66 kDa ERα, but instead a truncated 45 kDa ERα, we hypothesize that ER ligands might regulate TFPI expression through the truncated ERα with ligand specificity different from the classical, nuclear ERα. An additional interesting observation was that the expression of truncated 45 kDa ERα was not regulated by estrogens, SERMs or SERD.

## Competing interests

The author(s) declare that they have no competing interests.

## Authors' contributions

AD participated in the design of the study, the cell-culture experiments, interpretation of data, supervised the TFPI protein analysis, carried out the statistical analysis, and drafted the manuscript.

NI participated in the design of the study, supervised all aspects of the cell-culture experiments and the statistical analysis, participated in the interpretation of data, and helped drafting the manuscript.

BB participated in the cell-culture experiments, interpretation of data, and drafting of the manuscript.

AHR carried out the immunostaining analysis, and participated in interpretation of data, and drafting of the manuscript.

PMS participated in the design of the study, the interpretation of data, and drafting of the manuscript.

## Pre-publication history

The pre-publication history for this paper can be accessed here:



## References

[B1] Daly E, Vessey MP, Hawkins MM, Carson JL, Gough P, Marsh S (1996). Risk of venous thromboembolism in users of hormone replacement therapy. Lancet.

[B2] Grodstein F, Stampfer MJ, Goldhaber SZ, Manson JE, Colditz GA, Speizer FE, Willett WC, Hennekens CH (1996). Prospective study of exogenous hormones and risk of pulmonary embolism in women. Lancet.

[B3] Jick H, Derby LE, Myers MW, Vasilakis C, Newton KM (1996). Risk of hospital admission for idiopathic venous thromboembolism among users of postmenopausal oestrogens. Lancet.

[B4] Hoibraaten E, Qvigstad E, Arnesen H, Larsen S, Wickstrom E, Sandset PM (2000). Increased risk of recurrent venous thromboembolism during hormone replacement therapy--results of the randomized, double-blind, placebo-controlled estrogen in venous thromboembolism trial (EVTET). Thromb Haemost.

[B5] Koster T, Small RA, Rosendaal FR, Helmerhorst FM (1995). Oral contraceptives and venous thromboembolism: a quantitative discussion of the uncertainties. J Intern Med.

[B6] (1995). Venous thromboembolic disease and combined oral contraceptives: results of international multicentre case-control study. World Health Organization Collaborative Study of Cardiovascular Disease and Steroid Hormone Contraception. Lancet.

[B7] Cummings SR, Eckert S, Krueger KA, Grady D, Powles TJ, Cauley JA, Norton L, Nickelsen T, Bjarnason NH, Morrow M, Lippman ME, Black D, Glusman JE, Costa A, Jordan VC (1999). The effect of raloxifene on risk of breast cancer in postmenopausal women: results from the MORE randomized trial. Multiple Outcomes of Raloxifene Evaluation. JAMA.

[B8] Fisher B, Costantino JP, Wickerham DL, Redmond CK, Kavanah M, Cronin WM, Vogel V, Robidoux A, Dimitrov N, Atkins J, Daly M, Wieand S, Tan-Chiu E, Ford L, Wolmark N (1998). Tamoxifen for prevention of breast cancer: report of the National Surgical Adjuvant Breast and Bowel Project P-1 Study. J Natl Cancer Inst.

[B9] Rossouw JE, Anderson GL, Prentice RL, LaCroix AZ, Kooperberg C, Stefanick ML, Jackson RD, Beresford SA, Howard BV, Johnson KC, Kotchen JM, Ockene J (2002). Risks and benefits of estrogen plus progestin in healthy postmenopausal women: principal results From the Women's Health Initiative randomized controlled trial. JAMA.

[B10] Kemmeren JM, Tanis BC, van den Bosch MA, Bollen EL, Helmerhorst FM, van der Graaf Y, Rosendaal FR, Algra A (2002). Risk of Arterial Thrombosis in Relation to Oral Contraceptives (RATIO) study: oral contraceptives and the risk of ischemic stroke. Stroke.

[B11] Tanis BC, van den Bosch MA, Kemmeren JM, Cats VM, Helmerhorst FM, Algra A, van der Graaf Y, Rosendaal FR (2001). Oral contraceptives and the risk of myocardial infarction. N Engl J Med.

[B12] Hoibraaten E, Mowinckel MC, de Ronde H, Bertina RM, Sandset PM (2001). Hormone replacement therapy and acquired resistance to activated protein C: results of a randomized, double-blind, placebo-controlled trial. Br J Haematol.

[B13] Rosing J, Tans G (1999). Effects of oral contraceptives on hemostasis and thrombosis. Am J Obstet Gynecol.

[B14] Astrup T (1958). The haemostatic balance. Thromb Diath Haemorrh.

[B15] Rosendaal FR, van Hylckama Vlieg A, Tanis BC, Helmerhorst FM (2003). Estrogens, progestogens and thrombosis. J Thromb Haemost.

[B16] Robertson JF, Come SE, Jones SE, Beex L, Kaufmann M, Makris A, Nortier JW, Possinger K, Rutqvist LE (2005). Endocrine treatment options for advanced breast cancer--the role of fulvestrant. Eur J Cancer.

[B17] Bajaj MS, Kuppuswamy MN, Saito H, Spitzer SG, Bajaj SP (1990). Cultured normal human hepatocytes do not synthesize lipoprotein-associated coagulation inhibitor: evidence that endothelium is the principal site of its synthesis. Proc Natl Acad Sci U S A.

[B18] Broze GJJ (1995). Tissue factor pathway inhibitor and the revised theory of coagulation. Annu Rev Med.

[B19] Dahm A, van Hylckama Vlieg A, Bendz B, Rosendaal F, Bertina RM, Sandset PM (2003). Low levels of tissue factor pathway inhibitor (TFPI) increase the risk of venous thrombosis. Blood.

[B20] Morange PE, Simon C, Alessi MC, Luc G, Arveiler D, Ferrieres J, Amouyel P, Evans A, Ducimetiere P, Juhan-Vague I (2004). Endothelial cell markers and the risk of coronary heart disease: the Prospective Epidemiological Study of Myocardial Infarction (PRIME) study. Circulation.

[B21] Negaard H, Dahm A, Sandset PM, Iversen PO, Ostenstad B (2005). Angiogenesis and hemostasis in hematological neoplasias. Curr Drug Targets.

[B22] Mousa SA, Mohamed S (2004). Inhibition of endothelial cell tube formation by the low molecular weight heparin, tinzaparin, is mediated by tissue factor pathway inhibitor. Thromb Haemost.

[B23] Erman M, Abali H, Oran B, Haznedaroglu IC, Canpinar H, Kirazli S, Celik I (2004). Tamoxifen-induced tissue factor pathway inhibitor reduction: a clue for an acquired thrombophilic state?. Ann Oncol.

[B24] Hoibraaten E, Qvigstad E, Andersen TO, Mowinckel MC, Sandset PM (2001). The effects of hormone replacement therapy (HRT) on hemostatic variables in women with previous venous thromboembolism--results from a randomized, double-blind, clinical trial. Thromb Haemost.

[B25] Harris GM, Stendt CL, Vollenhoven BJ, Gan TE, Tipping PG (1999). Decreased plasma tissue factor pathway inhibitor in women taking combined oral contraceptives. Am J Hematol.

[B26] Jaffe EA, Nachman RL, Becker CG, Minick CR (1973). Culture of human endothelial cells derived from umbilical veins. Identification by morphologic and immunologic criteria. J Clin Invest.

[B27] Pfaffl MW, Horgan GW, Dempfle L (2002). Relative expression software tool (REST) for group-wise comparison and statistical analysis of relative expression results in real-time PCR. Nucleic Acids Res.

[B28] Bilsel AS, Onaran N, Moini H, Emerk K (2000). Long-term effect of 17beta-estradiol and thrombin on tissue factor pathway inhibitor release from HUVEC. Thromb Res.

[B29] Shirk RA, Zhang Z, Winneker RC (2005). Differential effects of estrogens and progestins on the anticoagulant tissue factor pathway inhibitor in the rat. J Steroid Biochem Mol Biol.

[B30] Simoncini T, Genazzani AR, Liao JK (2002). Nongenomic mechanisms of endothelial nitric oxide synthase activation by the selective estrogen receptor modulator raloxifene. Circulation.

[B31] Klinge CM, Blankenship KA, Risinger KE, Bhatnagar S, Noisin EL, Sumanasekera WK, Zhao L, Brey DM, Keynton RS (2005). Resveratrol and estradiol rapidly activate MAPK signaling through estrogen receptors alpha and beta in endothelial cells. J Biol Chem.

[B32] Savolainen-Peltonen H, Luoto NM, Kangas L, Hayry P (2004). Selective estrogen receptor modulators prevent neointima formation after vascular injury. Mol Cell Endocrinol.

[B33] Petit L, Lesnik P, Dachet C, Hugou I, Moreau M, Chapman J, Rouis M (1999). The promoter of human tissue factor pathway inhibitor gene: identification of potential regulatory elements. Thromb Res.

[B34] Flouriot G, Brand H, Denger S, Metivier R, Kos M, Reid G, Sonntag-Buck V, Gannon F (2000). Identification of a new isoform of the human estrogen receptor-alpha (hER-alpha) that is encoded by distinct transcripts and that is able to repress hER-alpha activation function 1. EMBO J.

[B35] Russell KS, Haynes MP, Sinha D, Clerisme E, Bender JR (2000). Human vascular endothelial cells contain membrane binding sites for estradiol, which mediate rapid intracellular signaling. Proc Natl Acad Sci U S A.

[B36] Figtree GA, McDonald D, Watkins H, Channon KM (2003). Truncated estrogen receptor alpha 46-kDa isoform in human endothelial cells: relationship to acute activation of nitric oxide synthase. Circulation.

[B37] Revankar CM, Cimino DF, Sklar LA, Arterburn JB, Prossnitz ER (2005). A transmembrane intracellular estrogen receptor mediates rapid cell signaling. Science.

[B38] Wijayaratne AL, McDonnell DP (2001). The human estrogen receptor-alpha is a ubiquitinated protein whose stability is affected differentially by agonists, antagonists, and selective estrogen receptor modulators. J Biol Chem.

[B39] Kaneko KJ, Furlow JD, Gorski J (1993). Involvement of the coding sequence for the estrogen receptor gene in autologous ligand-dependent down-regulation. Mol Endocrinol.

[B40] Ree AH, Landmark BF, Eskild W, Levy FO, Lahooti H, Jahnsen T, Aakvaag A, Hansson V (1989). Autologous down-regulation of messenger ribonucleic acid and protein levels for estrogen receptors in MCF-7 cells: an inverse correlation to progesterone receptor levels. Endocrinology.

[B41] Valley CC, Metivier R, Solodin NM, Fowler AM, Mashek MT, Hill L, Alarid ET (2005). Differential regulation of estrogen-inducible proteolysis and transcription by the estrogen receptor alpha N terminus. Mol Cell Biol.

[B42] McDonnell DP (2005). The molecular pharmacology of estrogen receptor modulators: implications for the treatment of breast cancer. Clin Cancer Res.

[B43] Richardson MA, Berg DT, Calnek DS, Ciaccia AV, Joyce DE, Grinnell BW (2000). 17beta-estradiol, but not raloxifene, decreases thrombomodulin in the antithrombotic protein C pathway. Endocrinology.

[B44] Smith LH, Coats SR, Qin H, Petrie MS, Covington JW, Su M, Eren M, Vaughan DE (2004). Differential and opposing regulation of PAI-1 promoter activity by estrogen receptor alpha and estrogen receptor beta in endothelial cells. Circ Res.

[B45] Madamanchi N, Niu XL, Runge MS (2004). A new slice of pie? Estrogen regulation of plasminogen activator inhibitor-1. Circ Res.

[B46] Cosman F, Baz-Hecht M, Cushman M, Vardy MD, Cruz JD, Nieves JW, Zion M, Lindsay R (2005). Short-term effects of estrogen, tamoxifen and raloxifene on hemostasis: a randomized-controlled study and review of the literature. Thromb Res.

